# Tigmint: correcting assembly errors using linked reads from large molecules

**DOI:** 10.1186/s12859-018-2425-6

**Published:** 2018-10-26

**Authors:** Shaun D. Jackman, Lauren Coombe, Justin Chu, Rene L. Warren, Benjamin P. Vandervalk, Sarah Yeo, Zhuyi Xue, Hamid Mohamadi, Joerg Bohlmann, Steven J.M. Jones, Inanc Birol

**Affiliations:** 1 BC Cancer Genome Sciences Centre, Vancouver, V5Z 4S6 BC Canada; 2 University of British Columbia, Michael Smith Laboratories, Vancouver, V6T 1Z4 BC Canada

**Keywords:** Assembly correction, Genome scaffolding, Genome sequence assembly, 10x Genomics Chromium, Linked reads

## Abstract

**Background:**

Genome sequencing yields the sequence of many short snippets of DNA (reads) from a genome. Genome assembly attempts to reconstruct the original genome from which these reads were derived. This task is difficult due to gaps and errors in the sequencing data, repetitive sequence in the underlying genome, and heterozygosity. As a result, assembly errors are common. In the absence of a reference genome, these misassemblies may be identified by comparing the sequencing data to the assembly and looking for discrepancies between the two. Once identified, these misassemblies may be corrected, improving the quality of the assembled sequence. Although tools exist to identify and correct misassemblies using Illumina paired-end and mate-pair sequencing, no such tool yet exists that makes use of the long distance information of the large molecules provided by linked reads, such as those offered by the 10x Genomics Chromium platform. We have developed the tool Tigmint to address this gap.

**Results:**

To demonstrate the effectiveness of Tigmint, we applied it to assemblies of a human genome using short reads assembled with ABySS 2.0 and other assemblers. Tigmint reduced the number of misassemblies identified by QUAST in the ABySS assembly by 216 (27%). While scaffolding with ARCS alone more than doubled the scaffold NGA50 of the assembly from 3 to 8 Mbp, the combination of Tigmint and ARCS improved the scaffold NGA50 of the assembly over five-fold to 16.4 Mbp. This notable improvement in contiguity highlights the utility of assembly correction in refining assemblies. We demonstrate the utility of Tigmint in correcting the assemblies of multiple tools, as well as in using Chromium reads to correct and scaffold assemblies of long single-molecule sequencing.

**Conclusions:**

Scaffolding an assembly that has been corrected with Tigmint yields a final assembly that is both more correct and substantially more contiguous than an assembly that has not been corrected. Using single-molecule sequencing in combination with linked reads enables a genome sequence assembly that achieves both a high sequence contiguity as well as high scaffold contiguity, a feat not currently achievable with either technology alone.

## Background

**Fig. 1:**
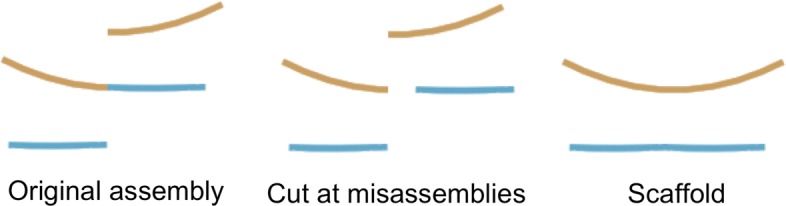
An assembly of a hypothetical genome with two linear chromosomes is assembled in three contigs. One of those contigs is misassembled. In its current misassembled state, this assembly cannot be completed by scaffolding alone. The misassembled contig must first be corrected by cutting the contig at the location of the misassembly. After correcting the missasembly, each chromosome may be assembled into a single scaffold

Assemblies of short read sequencing data are easily confounded by repetitive sequences larger than the fragment size of the sequencing library. When the size of a repeat exceeds the library fragment size, the contig comes to an end in the best case, or results in misassembled sequence in the worst case. Misassemblies not only complicate downstream analyses, but also limit the contiguity of the assembly. Each incorrectly assembled sequence prevents joining that chimeric sequence to its true neighbours during assembly scaffolding, illustrated in Fig. 1.

Long-read sequencing technologies have greatly improved assembly contiguity with their ability to span these repeats, but at a cost currently significantly higher than that of short-read sequencing technology. For population studies and when sequencing large genomes, such as conifer genomes and other economically important crop species, this cost may be prohibitive. The 10x Genomics (Pleasanton, CA) Chromium technology generates linked reads from large DNA molecules at a cost comparable to standard short-read sequencing technologies. Whereas paired-end sequencing gives two reads from a small DNA fragment, linked reads yield roughly a hundred read pairs from molecules with a typical size of a hundred kilobases. Linked reads indicate which reads were derived from the same DNA molecule (or molecules, when they share the same barcode), and so should be in close proximity in the underlying genome. The technology has been used previously to phase diploid genomes using a reference [1], *de novo* assemble complex genomes in the gigabase scale [2], and further scaffold draft assemblies [3, 4].

A number of software tools employ linked reads for various applications. The Long Ranger tool maps reads to repetitive sequence, phases small variants, and identifies structural variants [5], while Supernova [2] assembles diploid genome sequences. Both tools are developed by the vendor. Among tools from academic labs, GROC-SVs [6], NAIBR [7], and Topsorter [8] identify structural variants, and ARCS [4], Architect [9], and fragScaff [10] scaffold genome assemblies using linked reads.

In *de novo* sequencing projects, it is challenging yet important to ensure the correctness of the resulting assemblies. Tools to correct misassemblies typically inspect the reads aligned back to the assembly to identify discrepancies. Pilon [11] inspects the alignments to identify variants and correct small-scale misassemblies. NxRepair [12] uses Illumina mate-pair sequencing to correct large-scale structural misassemblies. Misassemblies may also be corrected using optical mapping and chromosome conformation capture [13]. Linked reads offer an opportunity to use the long-range information provided by large molecules to identify misassemblies in a cost-effective way, yet no software tool currently exists to correct misassemblies using linked reads. Here we introduce a software tool, Tigmint, to identify misassemblies using this new and useful data type.

Tigmint first aligns linked reads to an assembly, and infers the extents of the large DNA molecules from these alignments. It then searches for atypical drops in physical molecule coverage, revealing the positions of possible misassemblies. It cuts the assembled sequences at these positions to improve assembly correctness. Linked reads may then be used again to scaffold the corrected assembly with ARCS [4] to identify contig ends sharing barcodes, and either ABySS-Scaffold (included with ABySS) or LINKS [14] to merge sequences of contigs into scaffolds.

## Methods

**Fig. 2:**
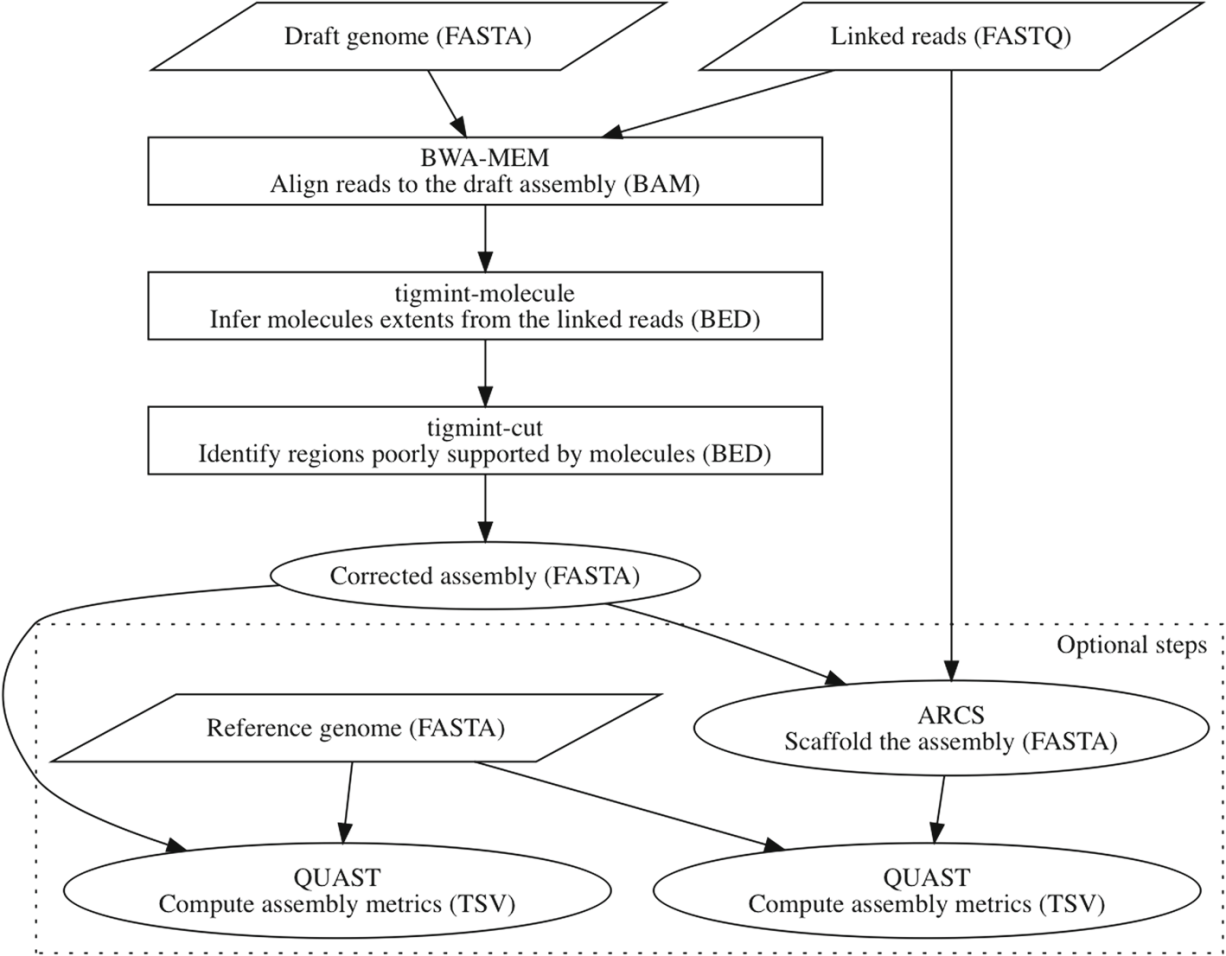
The block diagram of Tigmint. Input files are shown in parallelograms. Intermediate files are shown in rectangles. Output files are shown in ovals. File formats are shown in parentheses

Tigmint identifies misassembled regions of the assembly by inspecting the alignment of linked reads to the draft genome assembly. The command tigmint-molecule groups linked reads with the same barcode into molecules. The command tigmint-cut identifies regions of the assembly that are not well supported by the linked reads, and cuts the contigs of the draft assembly at these positions. Tigmint may optionally scaffold the genome using ARCS [4]. A block diagram of the analysis pipeline is shown in Fig. 2.

A typical workflow of Tigmint is as follows. The user provides a draft assembly in FASTA format and the linked reads in FASTQ format. Tigmint first aligns the linked reads to the draft genome using BWA-MEM [15]. The alignments are filtered by alignment score and number of mismatches to remove poorly aligned reads with the default thresholds NM<5 and AS≥0.65·*l*, where *l* is the read length. Reads with the same barcode that map within a specified distance, 50 kbp by default, of the adjacent reads are grouped into a molecule. A BED (Browser Extensible Data) file [16] is constructed, where each record indicates the start and end of one molecule, and the number of reads that compose that molecule. Unusually small molecules, shorter than 2 kbp by default, are filtered out.

Physical molecule depth of coverage is the number of molecules that span a point. A molecule spans a point when one of its reads aligns to the left of that point and another of its reads (with the same barcode) aligns to the right of that point. Regions with poor physical molecule coverage indicate potentially problematic regions of the assembly. At a misassembly involving a repeat, molecules may start in left flanking unique sequence and end in the repeat, and molecules may start in the repeat and end in right flanking unique sequence. This seemingly uninterrupted molecule coverage may give the appearance that the region is well covered by molecules. Closer inspection may reveal that no molecules span the repeat entirely, from the left flanking sequence to the right flanking sequence. Tigmint checks that each region of a fixed size specified by the user, 1000 bp by default, is spanned by a minimum number of molecules, 20 by default.

Tigmint constructs an interval tree of the coordinates of the molecules using the Python package Intervaltree. The interval tree allows us to quickly identify and count the molecules that span a given region of the draft assembly. Regions that have a sufficient number of spanning molecules, 20 by default, are deemed well-covered, and regions that do not are deemed poorly-covered and reveal possible misassemblies. We inspect the molecule coverage of each contig with a sliding window of 1000 bp (by default) with a step size of 1 bp. Tigmint cuts the assembly after the last base of a well-covered window before a run of poorly-covered windows, and then cut the assembly again before the first base of the first well-covered window following that run of poorly-covered windows, shown in Listing 1. The coordinates of these cut points are recorded in a BED file. The sequences of the draft assembly are split at these cut points, producing a corrected FASTA file.


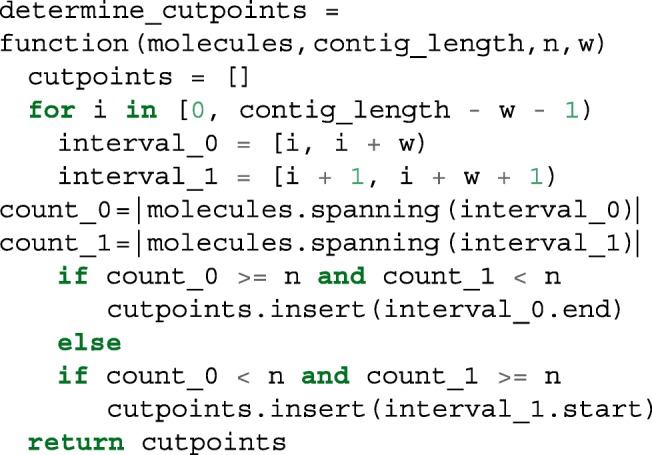


Tigmint will optionally run ARCS [4] to scaffold these corrected sequences and improve the contiguity of the assembly. Tigmint corrects misassemblies in the draft genome to improve the correctness of the assembly, but Tigmint itself cannot improve the contiguity of the assembly. ARCS merges contigs into scaffolds by identifying ends of contigs that share common barcodes. However, ARCS in itself would not be able to make the join if the correct mate of a contig end is buried deep within a misassembled contig. Tigmint corrects the misassembly, which exposes the end of the previously misassembled contig, so that ARCS is now able to make that merge. Tigmint and ARCS work together to improve both the correctness and contiguity of an assembly.

Tigmint will optionally compare the scaffolds to a reference genome, if one is provided, using QUAST [17] to compute contiguity (NGA50) and correctness (number of putative misassemblies) of the assemblies before Tigmint, after Tigmint, and after ARCS. Each misassembly identified by QUAST reveals a difference between the assembly and the reference, and may indicate a real misassembly or a structural variation between the reference and the sequenced genome. The NGA50 metric summarizes both assembly contiguity and correctness by computing the NG50 of the lengths of alignment blocks to a reference genome, correcting the contiguity metric by accounting for possible misassemblies. It however also penalizes sequences at points of true variation between the sequenced and reference genomes. The true but unknown contiguity of the assembly, which accounts for misassemblies but not for structural variation, therefore lies somewhere between the lower bound of NGA50 and the upper bound of NG50.

## Evaluation

We have evaluated the effectiveness of Tigmint on assemblies of both short and long read sequencing data, including assemblies of Illumina paired-end and mate-pair sequencing using ABySS and DISCOVARdenovo, a Supernova assembly of linked reads, a Falcon assembly of PacBio sequencing, a Canu assembly of Oxford Nanopore sequencing, and an ABySS assembly of simulated Illumina sequencing (see Table 1). All assemblies are of the Genome in a Bottle (GIAB) human sample HG004, except the Canu assembly of human sample NA12878. The sample HG004 was selected for the variety of data types available, including Illumina 2x250 paired-end and mate-pair sequencing, linked reads, and PacBio sequencing [18]. NA12878 was selected for the availability of an assembly of Oxford Nanopore sequencing [19] as well as the linked read sequencing needed by Tigmint.

**Table 1:** Genome assemblies of both short and long read sequencing were used to evaluate Tigmint

Sample	Sequencing Platform	Assembler
HG004	Illumina	ABySS
HG004	Illumina	DISCOVARdenovo
HG004	10x Chromium	Supernova
HG004	PacBio	Falcon
NA12878	Oxford Nanopore	Canu

The GIAB sample HG004 is also known as NA24143. See “Availability of data and material” to access the sequencing data and assemblies

We downloaded the ABySS 2.0 [20] assembly of HG004 abyss-2.0/scaffolds.fa from NCBI, assembled from Illumina paired-end and mate-pair reads [18]. We downloaded the Illumina mate pair reads for this individual from NCBI. We trimmed adapters using NxTrim 0.4.0 [21] with parameters --norc --joinreads --preserve-mp and selected the reads identified as known mate pairs. We ran NxRepair 0.13 [12] to correct the ABySS 2.0 assembly of HG004 using these trimmed mate-pair reads. A range of values of its z-score threshold parameter *T* were tested.

We downloaded the 10x Genomics Chromium reads for this same individual from NCBI, and we extracted barcodes from the reads using Long Ranger Basic. We ran Tigmint to correct the ABySS 2.0 assembly of HG004 using these Chromium reads with the parameters window = 2000 and span = 20. The choice of parameters is discussed in the results. Both the uncorrected and corrected assemblies were scaffolded using ARCS. These assemblies were compared to the chromosome sequences of the GRCh38 reference genome using QUAST [17]. Since ARCS version 1.0.0 that we used does not estimate gap sizes using linked reads, the QUAST parameter --scaffold-gap-max-size is set to 100 kbp.

We repeated this analysis using Tigmint, ARCS, and QUAST with five other assemblies. We downloaded the reads assembled with DISCOVARdenovo and scaffolded using BESST [22] from NCBI, and the same DISCOVARdenovo contigs scaffolded using ABySS-Scaffold. We assembled the linked reads with Supernova 2.0.0 [2], which used neither the 2 ×250 paired-end reads nor mate-pair reads.

We applied Tigmint and ARCS to two assemblies of single-molecule sequencing (SMS) reads. We downloaded PacBio reads assembled with Falcon from NCBI [23] and Oxford Nanopore reads assembled with Canu [19].

Most software used in these analyses were installed using Linuxbrew [24] with the command brew tap brewsci/bio; brew install abyss arcs bwa lrsim miller minimap2 nxtrim samtools seqtk. We used the development version of QUAST 5 revision 78806b2, which is capable of analyzing assemblies of large genomes using Minimap2 [25].

## Results and discussion

**Fig. 3:**
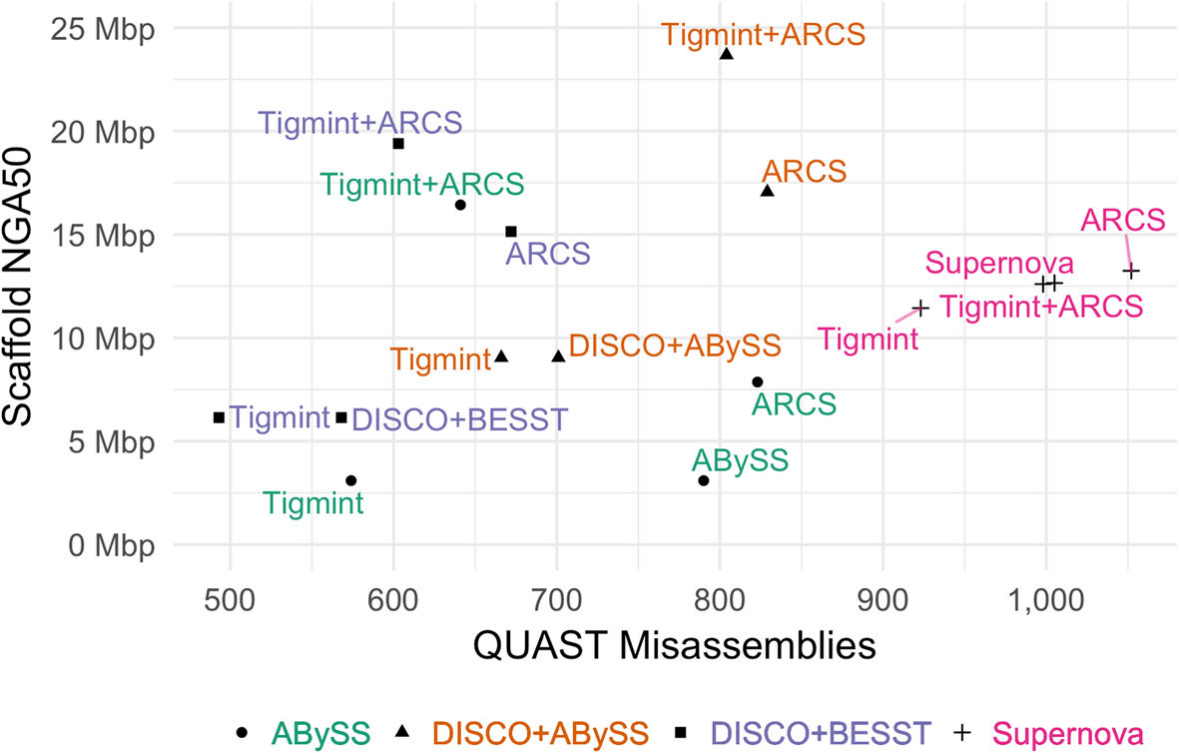
Assembly contiguity and correctness metrics of HG004 with and without correction using Tigmint prior to scaffolding with ARCS. The most contiguous and correct assemblies are found in the top-left. Supernova assembled linked reads only, whereas the others used paired end and mate pair reads

Correcting the ABySS assembly of the human data set HG004 with Tigmint reduces the number of misassemblies identified by QUAST by 216, a reduction of 27%. While the scaffold NG50 decreases slightly from 3.65 Mbp to 3.47 Mbp, the scaffold NGA50 remains unchanged; thus in this case, correcting the assembly with Tigmint improves the correctness of the assembly without substantially reducing its contiguity. However, scaffolding the uncorrected and corrected assemblies with ARCS yield markedly different results: a 2.5-fold increase in NGA50 from 3.1 Mbp to 7.9 Mbp without Tigmint versus a more than five-fold increase in NGA50 to 16.4 Mbp with Tigmint. Further, correcting the assembly and then scaffolding yields a final assembly that is both more correct and more contiguous than the original assembly, as shown in Fig. 3 and Table 2.

**Table 2:** The assembly contiguity (scaffold NG50 and NGA50) and correctness (number of misassemblies) metrics with and without correction using Tigmint prior to scaffolding with ARCS

Sample	Assembly	NG50 (Mbp)	NGA50 (Mbp)	Misass.	Reduction
HG004	ABySS	3.65	3.09	790	NA
	ABySS+Tigmint	3.47	3.09	574	216 (27.3%)
	ABySS+ARCS	9.91	7.86	823	NA
	ABySS+Tigmint+ARCS	26.39	16.43	641	182 (22.1%)
HG004	DISCO+ABySS	10.55	9.04	701	NA
	DISCO+ABySS+Tigmint	10.16	9.04	666	35 (5.0%)
	DISCO+ABySS+ARCS	29.20	17.05	829	NA
	DISCO+ABySS+Tigmint+ARCS	35.31	23.68	804	25 (3.0%)
HG004	DISCO+BESST	7.01	6.14	568	NA
	DISCO+BESST+Tigmint	6.77	6.14	493	75 (13.2%)
	DISCO+BESST+ARCS	27.64	15.14	672	NA
	DISCO+BESST+Tigmint+ARCS	33.43	19.40	603	69 (10.3%)
HG004	Supernova	38.48	12.65	1005	NA
	Supernova+Tigmint	17.72	11.43	923	82 (8.2%)
	Supernova+ARCS	39.63	13.24	1052	NA
	Supernova+Tigmint+ARCS	27.35	12.60	998	54 (5.1%)
HG004	Falcon	4.56	4.21	3640	NA
	Falcon+Tigmint	4.45	4.21	3444	196 (5.4%)
	Falcon+ARCS	18.14	9.71	3,801	NA
	Falcon+Tigmint+ARCS	22.52	11.97	3,574	227 (6.0%)
NA12878	Canu	7.06	5.40	1688	NA
	Canu+Tigmint	6.87	5.38	1600	88 (5.2%)
	Canu+ARCS	19.70	10.12	1736	NA
	Canu+Tigmint+ARCS	22.01	10.85	1,626	110 (6.3%)
Simulated	ABySS	9.00	8.28	272	NA
	ABySS+Tigmint	8.61	8.28	217	55 (20.2%)
	ABySS+ARCS	23.37	17.09	365	NA
	ABySS+Tigmint+ARCS	30.24	24.98	320	45 (12.3%)

ABySS and DISCOVARdenovo are assemblies of Illumina sequencing. Supernova is an assembly of linked read sequencing. Falcon is an assembly of PacBio sequencing. Canu is an assembly Oxford Nanopore sequencing. Data simulated with LRSim is assembled with ABySS

Correcting the DISCOVARdenovo + BESST assembly reduces the number of misassemblies by 75, a reduction of 13%. Using Tigmint to correct the assembly before scaffolding with ARCS yields an increase in NGA50 of 28% over using ARCS without Tigmint. Correcting the DISCOVARdenovo + ABySS-Scaffold assembly reduces the number of misassemblies by 35 (5%), after which scaffolding with ARCS improves the NGA50 to 23.7 Mbp, 2.6 times the original assembly and a 40% improvement over ARCS without Tigmint. The assembly with the fewest misassemblies is DISCOVARdenovo + BESST + Tigmint. The assembly with the largest NGA50 is DISCOVARdenovo + ABySS-Scaffold + Tigmint + ARCS. Finally, DISCOVARdenovo + BESST + Tigmint + ARCS strikes a good balance between both good contiguity and few misassemblies.

Correcting the Supernova assembly of the HG004 linked reads with Tigmint reduces the number of misassemblies by 82, a reduction of 8%, and after scaffolding the corrected assembly with ARCS, we see a slight (<1%) decrease in both misassemblies and NGA50 compared to the original Supernova assembly. Since the Supernova assembly is composed entirely of the linked reads, this result is concordant with our expectation of no substantial gains from using these same data to correct and scaffold the Supernova assembly.

**Fig. 4:**
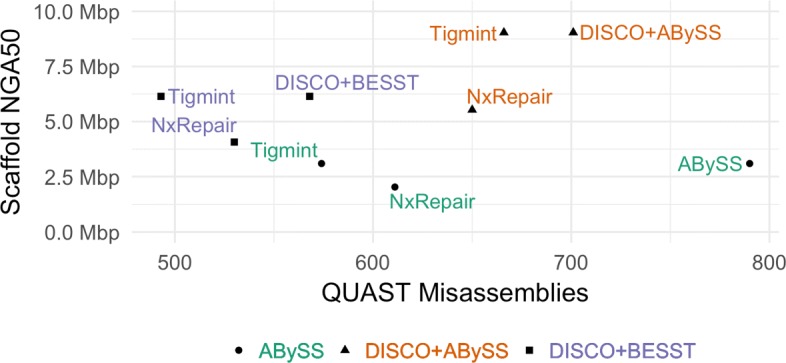
Assembly contiguity and correctness metrics of HG004 corrected with NxRepair, which uses mate pairs, and Tigmint, which uses linked reads. The most contiguous and correct assemblies are found in the top-left

We attempted to correct the ABySS assembly using NxRepair, which made no corrections for any value of its z-score threshold parameter *T* less than -2.7. Setting *T* = -2.4, NxRepair reduced the number of misassemblies from 790 to 611, a reduction of 179 or 23%, whereas Tigmint reduced misassemblies by 216 or 27%. NxRepair reduced the NGA50 by 34% from 3.09 Mbp to 2.04 Mbp, unlike Tigmint, which did not reduce the NGA50 of the assembly. Tigmint produced an assembly that is both more correct and more contiguous than NxRepair with *T* = -2.4. Smaller values of *T* corrected fewer errors than Tigmint, and lager values of *T* further decreased the contiguity of the assembly. We similarly corrected the two DISCOVARdenovo assemblies using NxRepair with *T* = -2.4, shown in figure Fig. 4. The DISCOVARdenovo + BESST assembly corrected by Tigmint is both more correct and more contiguous than that corrected by NxRepair. The DISCOVARdenovo + ABySS-Scaffold assembly corrected by NxRepair has 16 (2.5%) fewer misassemblies than that corrected by Tigmint, but the NGA50 is reduced from 9.04 Mbp with Tigmint to 5.53 Mbp with NxRepair, a reduction of 39%.

**Fig. 5:**
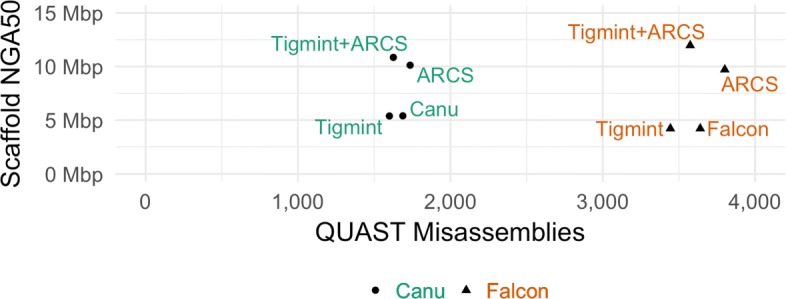
Assemblies of Oxford Nanopore sequencing of NA12878 with Canu and PacBio sequencing of HG004 with Falcon with and without correction using Tigmint prior to scaffolding with ARCS

The assemblies of SMS reads have contig NGA50s in the megabases. Tigmint and ARCS together improve the scaffold NGA50 of the Canu assembly by more than double to nearly 11 Mbp and improve the scaffold NGA50 of the Falcon assembly by nearly triple to 12 Mbp, and both assemblies have fewer misassemblies than their original assembly, shown in Fig. 5. Thus, using Tigmint and ARCS together improves both the contiguity and correctness over the original assemblies. This result demonstrates that by using long reads in combination with linked reads, one can achieve an assembly quality that is not currently possible with either technology alone.

**Fig. 6:**
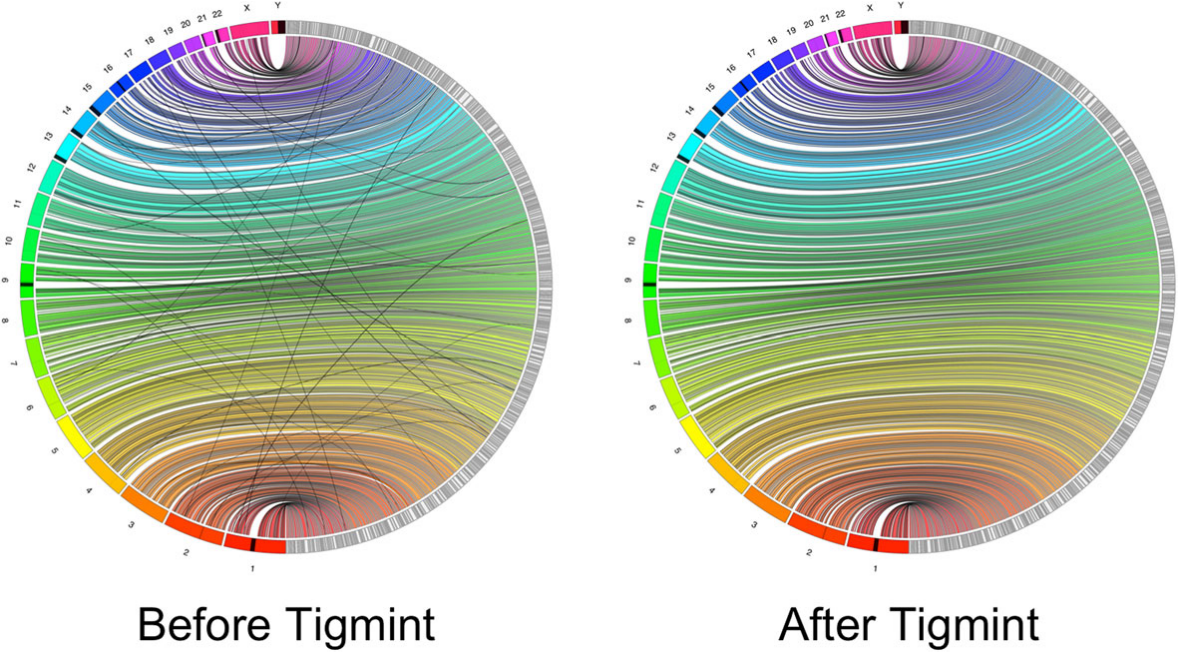
The alignments to the reference genome of the ABySS assembly of HG004 before and after Tigmint. The reference chromosomes are on the left in colour, the assembly scaffolds on the right in grey. No translocations are visible after Tigmint

The alignments of the ABySS assembly to the reference genome before and after Tigmint are visualized in Fig. 6 using JupiterPlot [26], which uses Circos [27]. A number of split alignments, likely misassemblies, are visible in the assembly before Tigmint, whereas after Tigmint no such split alignments are visible.

**Fig. 7:**
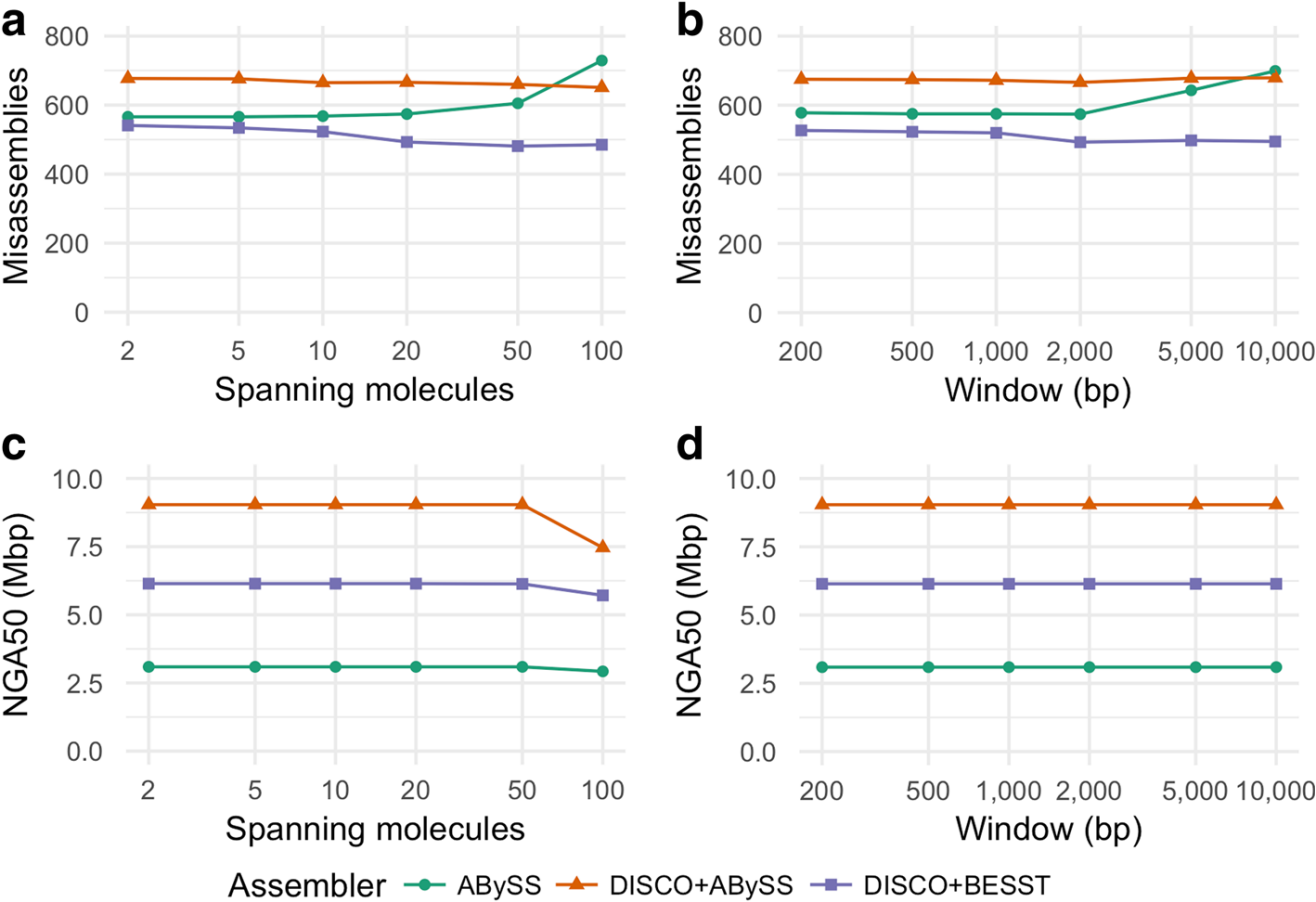
**a**. **b**. **c**. **d**. Effect of varying the window and span parameters on scaffold NGA50 and misassemblies of three assemblies of HG004

The default maximum distance permitted between linked reads in a molecule is 50 kbp, which is the value used by the Long Ranger and Lariat tools of 10x Genomics. In our tests, values between 20 kbp and 100 kbp do not substantially affect the results, and values smaller than 20 kbp begin to disconnect linked reads that should be found in a single molecule. The effect of varying the window and spanning molecules parameters of Tigmint on the assembly contiguity and correctness metrics is shown in Fig. 7. When varying the spanning molecules parameter, the window parameter is fixed at 2 kbp, and when varying the window parameter, the spanning molecules parameter is fixed at 20. The assembly metrics of the ABySS, DISCOVARdenovo + ABySS-Scaffold, and DISCOVARdenovo + BESST assemblies after correction with Tigmint are rather insensitive to the spanning molecules parameter for any value up to 50 and for the window parameter for any value up to 2 kbp. The parameter values of span=20 and window=2000 worked well for all of the tested assembly tools.

We simulated 434 million 2 ×250 paired-end and 350 million 2 ×125 mate-pair read pairs using wgsim of samtools, and we simulated 524 million 2 ×150 linked read pairs using LRSim [28], emulating the HG004 data set. We assembled these reads using ABySS 2.0.2, and applied Tigmint and ARCS as before. The assembly metrics are shown in Table 2. We see similar performance to the real data: a 20% reduction in misassemblies after running Tigmint, and a three-fold increase in NGA50 after Tigmint and ARCS. Since no structural rearrangements are present in the simulated data, each misassembly identified by QUAST ought to be a true misassembly, allowing us to calculate precision and recall. For the parameters used with the real data, window = 2000 and span = 20, Tigmint makes 210 cuts in scaffolds at least 3 kbp (QUAST does not analyze shorter scaffolds), and corrects 55 misassemblies of the 272 identified by QUAST, yielding precision and recall of $\text {PPV} = \frac {55}{210} = 0.26$ and $\text {TPR} = \frac {55}{272} = 0.20$. Altering the window parameter to 1 kbp, Tigmint makes only 58 cuts, and yet it corrects 51 misassemblies, making its precision and recall $\text {PPV} = \frac {51}{58} = 0.88$ and $\text {TPR} = \frac {51}{272} = 0.19$, a marked improvement in precision with only a small decrease in recall. The scaffold NGA50 after ARCS is 24.7 Mbp, 1% less than with window = 2000. Since the final assembly metrics are similar, using a smaller value for the window size parameter may avoid unnecessary cuts. Small-scale misassemblies cannot be detected by Tigmint, such as collapsed repeats, and relocations and inversions smaller than a typical molecule.

The primary steps of running Tigmint are mapping the reads to the assembly, determining the start and end coordinate of each molecule, and finally identifying the discrepant regions and correcting the assembly. Mapping the reads to the DISCOVAR + ABySS-Scaffold assembly with BWA-MEM and concurrently sorting by barcode using Samtools [29] in a pipe required 5.5 h (wall-clock) and 17.2 GB of RAM (RSS) using 48 threads on a 24-core hyper-threaded computer. Determining the start and end coordinates of each molecule required 3.25 h and 0.08 GB RAM using a single thread. Finally, identifying the discrepant regions of the assembly, correcting the assembly, and creating a new FASTA file required 7 min and 3.3 GB RAM using 48 threads. The slowest step of mapping the reads to the assembly could be made faster by using light-weight mapping rather than full alignment, since Tigmint needs only the positions of the reads, not their alignments. NxRepair required 74.9 GB of RAM (RSS) and 5h 19m of wall clock time using a single CPU core, since it is not parallelized.

When aligning an assembly of an individual’s genome to a reference genome of its species, we expect to see breakpoints where the assembled genome differs from the reference genome. These breakpoints are caused by both misassemblies and true differences between the individual and the reference. The median number of mobile-element insertions for example, just one class of structural variant, is estimated to be 1218 per individual [30]. Misassemblies can be corrected by inspecting the alignments of the reads to the assembly and cutting the scaffolds at positions not supported by the reads. Reported misassemblies due to true structural variation will however remain. For this reason, even a perfectly corrected assembly is expected to have a number of differences when compared to the reference.

## Conclusions

Tigmint uses linked reads to reduce the number of misassemblies in a genome sequence assembly. The contiguity of the assembly is not appreciably affected by such a correction, while yielding an assembly that is more correct. Most scaffolding tools order and orient the sequences that they are given, but do not attempt to correct misassemblies. These misassemblies hold back the contiguity that can be achieved by scaffolding. Two sequences that should be connected together cannot be when one of those two sequences is connected incorrectly to a third sequence. By first correcting these misassemblies, the scaffolding tool can do a better job of connecting sequences, and we observe precisely this synergistic effect. Scaffolding an assembly that has been corrected with Tigmint yields a final assembly that is both more correct and substantially more contiguous than an assembly that has not been corrected.

Linked read sequencing has two advantages over paired-end and mate-pair reads to identify and correct misassemblies. Firstly, the physical coverage of the large molecules of linked reads is more consistent and less prone to coverage dropouts than that of paired-end and mate-pair sequencing data. Since roughly a hundred read pairs are derived from each molecule, the mapping of the large molecule as a whole to the draft genome is less affected by the GC content and repetitiveness of any individual read. Secondly, paired-end and mate-pair reads are derived from molecules typically smaller than 1 kbp and 10 kbp respectively. Short reads align ambiguously to repetitive sequence that is larger than the DNA molecule size of the sequencing library. The linked reads of 10 × Genomics Chromium are derived from molecules of about 100 kbp, which are better able to uniquely align to repetitive sequence and resolve misassemblies around repeats.

Using single-molecule sequencing in combination with linked reads enables a genome sequence assembly that achieves both a high sequence contiguity as well as high scaffold contiguity, a feat not currently achievable with either technology alone. Although paired-end and mate-pair sequencing is often used to polish a long-read assembly to improve its accuracy at the nucleotide level, it is not well suited to polish the repetitive sequence of the assembly, where the reads align ambiguously. Linked reads would resolve this mapping ambiguity and are uniquely suited to polishing an assembly of long reads, an opportunity for further research in the hybrid assembly of long and linked reads.

## Availability and requirements

**Project name:** Tigmint

**Project home page:** https://github.com/bcgsc/tigmint

**Operating system:** Platform independent

**Programming language:** Python

**License:** GNU GPL v3.0

## Abbreviations

BED: Browser extensible data
bp: Base pair
GIAB: Genome in a bottle
kbp: Kilobase pair
NCBI: National center for biotechnology information
RAM: Random access memory
RSS: Resident set size
SMS: Single-molecule sequencing
SRA: Sequence read archive
